# Polysomnographic Analysis of Sleep-Disordered Breathing In Children With Mucopolysaccharidoses in Qatar: A Retrospective Study

**DOI:** 10.7759/cureus.79773

**Published:** 2025-02-27

**Authors:** Nadine Asir, Amal R Al-Naimi

**Affiliations:** 1 Pediatric Pulmonology, Sidra Medicine, Doha, QAT; 2 Pediatric Pulmonary, Sidra Medicine, Doha, QAT

**Keywords:** mucopolysaccharidoses, obstructive sleep apnea, polysomnography, sleep disordered breathing, snoring

## Abstract

Introduction: Sleep-disordered breathing (SDB) represents a critical complication in mucopolysaccharidoses (MPSs), with a reported global prevalence rate of 80%-90%. The multifactorial pathophysiology involves glycosaminoglycan deposition in upper airway tissues and complex skeletal alterations. Although SDB is frequently encountered among MPS patients, details on its prevalence and characteristics remain unknown in Qatar. This study highlights different aspects of SDB in our population.

Methods: A retrospective chart review study was conducted on patients with a confirmed diagnosis of MPS aged one to 18 between September 2019 and July 2023 who underwent polysomnography (PSG) study at Sidra Medicine (Doha, Qatar). Clinical and PSG data were collected and summarized.

Results: The cohort comprised 14 patients (50% male; mean age 8.2 years (range: 1.42-18.8); mean BMI 21.63 kg/m^2^ (range: 13.47-42.1)). Obstructive sleep apnea (OSA) prevalence was 78.57% (11/14), with severity distribution as follows: mild (27.27%), moderate (27.27%), and severe (45.45%). Mean obstructive apnea-hypopnea index (OAHI) was 13.48 events/hour. Therapeutic interventions included adenotonsillectomy (eight of 14) and non-invasive positive airway pressure support (six of 14). None of the patients required tracheostomy.

Conclusion: This first comprehensive analysis of SDB in Qatar's pediatric MPS population reveals high prevalence and severity rates comparable to international cohorts. These results emphasize the crucial need for polysomnographic screening and early therapeutic intervention in this population. Future prospective studies should evaluate short and long-term treatment outcomes and explore potential genotype-phenotype correlations in this demographically distinct cohort.

## Introduction

Mucopolysaccharidosis (MPS) comprises a diverse group of inherited lysosomal storage disorders with seven distinct types, most of which follow an autosomal recessive pattern [1]. With an estimated incidence of approximately one in 25,000 live births [2], MPS is characterized by deficiencies in specific enzymes that lead to the accumulation of glycosaminoglycans (GAGs) in various tissues. This pathological buildup adversely affects multiple organs and systems - including the lungs, airways, and skeleton - resulting in a wide range of clinical manifestations that vary from mild to severe [2,3]. Among the complications associated with MPS, sleep-disordered breathing (SDB) is particularly concerning. SDB in MPS patients encompasses a spectrum of conditions, ranging from simple snoring to increased airway resistance, and can progress to obstructive sleep apnea (OSA), a condition affecting more than 80% of patients [4]. In addition to OSA, hypoventilation and central apnea have also been documented [5]. The multifactorial etiology of SDB in this population is highlighted by GAG deposition in tissues such as the tongue, adenoids, pharynx, larynx, and tracheal wall, all contributing to airway narrowing [6]. Structural deformities in the spine and chest wall can further precipitate restrictive lung disease and respiratory insufficiency, thereby impairing gas exchange and ventilation [5]. Moreover, enlargement of the abdominal organs may restrict diaphragmatic movement, reducing overall ventilatory capacity [7].

Early recognition and treatment of SDB are paramount, given its association with significant morbidity and mortality. Untreated OSA increases the risk of systemic and pulmonary hypertension, heart failure, arrhythmia, and neurocognitive impairment [8]. In pediatric patients with MPS, polysomnography (PSG) remains the gold-standard diagnostic tool for the accurate identification of SDB.

Managing SDB in MPS patients is challenging and necessitates a multidisciplinary approach. While adenotonsillectomy may alleviate symptoms of upper airway obstruction, there is a high risk of recurrence [9]. Consequently, many patients require non-invasive positive airway pressure support, with tracheostomy reserved for severe cases. Additionally, enzyme replacement therapy has demonstrated potential in ameliorating SDB symptoms in this patient group [4,10].

In light of these challenges, our study aims to identify the prevalence and characteristics of SDB among MPS patients in Qatar and to improve the understanding of respiratory complications and guide the development of more effective diagnostic and therapeutic strategies in this population.

## Materials and methods

Patients and data

A retrospective chart review was conducted on patients with a confirmed diagnosis of MPS who underwent PSG at Sidra Medicine (Doha, Qatar) between September 2019 and July 2023. Eligible patients were between one and 18 years of age at the time of their first PSG. Patients were excluded if they had sleep efficiency lower than 40% or if their PSG data were incomplete. A total of 14 patients with three different types of MPS (type I, type IV, and type VI) were included in the study.

Patients were identified using the hospital’s electronic information system by searching for the diagnosis code of MPS. The system includes codes for various types of MPS: MPS type I (Hurler syndrome), MPS type II (Hunter syndrome), MPS type III (Sanfilippo syndrome), MPS type IV (Morquio syndrome), MPS type VI (Maroteaux-Lamy syndrome), MPS type VII (Sly syndrome), and MPS type IX (Hyaluronidase deficiency).

The clinical notes of these patients were retrospectively reviewed. Demographic data (age, gender, body mass index (BMI)) and clinical parameters (MPS type, symptoms of snoring and/or witnessed sleep apnea, comorbid medical conditions such as heart disease and scoliosis, presence of adeno-tonsillar hypertrophy, and history of adenotonsillectomy or tracheostomy) were extracted from the medical records.

Polysomnography

Each patient underwent an overnight PSG without sedation, following standard recommendations to evaluate SDB. The PSG studies were performed in the only pediatric sleep laboratory in the state of Qatar. During the study, a PSG technician observed the patient’s recordings, and all studies were subsequently scored by our sleep lab technician in accordance with the American Academy of Sleep Medicine (AASM) scoring criteria. A certified pulmonologist reviewed and reported the findings. A standard overnight PSG with the Philips Respironics Inc. system (Florida, USA) recorded various physiological parameters. Brain and muscle activity were monitored using an electroencephalogram (EEG), electrooculograms (EOG), and an electromyogram (EMG). Heart function was assessed through an electrocardiogram (ECG) and pulse rate measurement. Breathing patterns were tracked by measuring thoracic and abdominal movements with respiratory inductance plethysmography (RIP), while airflow was recorded using a nasal cannula with nasal pressure sensors. Oxygen levels (SpO₂) were continuously monitored with pulse oximetry, and transcutaneous CO₂ levels were documented when available. Additionally, limb movements, body posture, and video recordings were included to provide a comprehensive evaluation of the sleep study.

The following data were extracted from the sleep reports: sleep efficiency, sleep latency, rapid eye movement (REM) latency, wakefulness after sleep onset (WASO), REM %, apnea-hypopnea index (AHI), REM AHI, central apnea index, obstructive AHI (OAHI), average oxygen saturation, minimal oxygen saturation, percentage of total sleep time that was spent with oxygen saturation below 90%, and carbon dioxide level. Also, we identified the presence of snoring and paradoxical breathing.

OSA was defined as OAHI ≥ 1.5 events/hour. Severity of OSA was considered mild if OAHI was 1.5-4.9 events/hour, moderate if OAHI was 5-9.9 events/hour, and severe if OAHI was ≥ 10 events/hour. Central sleep apnea was considered present if the patient had central apnea events ≥ 5/hour [11]. Diagnosis of hypoventilation was considered if transcutaneous CO_2_ was > 50 mmHg for more than 25% of the total sleep time [12].

Data management

All extracted parameters were systematically coded and organized into columns within an Excel spreadsheet (Microsoft Corporation, Washington, DC, USA). Scores were entered under each respective column, and the mean and range of the PSG parameters were calculated using Excel’s formula functions.

Ethical considerations

The study was approved by the Sidra Medicine Institutional Review Board (IRBNet # 2082894). Consent from patients and parents was waived due to the retrospective nature of the study.

## Results

A total of 14 patients (seven males and seven females) with confirmed diagnosis of MPSs were identified and included in the study. Nine of the patients had Morquio syndrome (64.28%), three patients had Hurler syndrome (21.42%), and two patients had Maroteaux-Lamy syndrome (14.28%). The mean (range) age at the time of PSG study was 8.2 years (1.42-18.8). Mean (range) BMI was 21.63 kg/m^2^ (13.47-42.1).

The majority of patients were symptomatic (11 patients, 78.57%). Specifically, five patients (35.7%) had a history of snoring only, while six patients (42.85%) exhibited both snoring and witnessed sleep apnea. Regarding comorbidities, nine patients (64.28%) had cardiac abnormalities, four (28.57%) had scoliosis, and three (21.42%) had kyphoscoliosis. Data on adeno-tonsillar hypertrophy were unavailable in three charts; however, among the remaining patients, 81.81% presented with adeno-tonsillar hypertrophy. Detailed demographic and clinical characteristics are summarized in Table 1.

**Table 1:** Clinical characteristics of the study participants (n=14) PSG: polysomnography, BMI: body mass index, n: number of patients

Characteristics	
Age at time of PSG study in years mean (range)	8.2 (1.42 – 18.8)
Male, n (%)	7 (50)
Female, n (%)	7 (50)
BMI kg/m^2^, mean (range)	21.63 (13.47 – 42.1)
Morquio syndrome, n (%)	9 (64.28)
Hurler's syndrome, n (%)	3 (21.42)
Maroteaux-Lamy syndrome, n (%)	2 (14.28)
Only snoring, n (%)	5 (35.7)
Only witnessed sleep apnea, n (%)	0 (0)
Snoring and witnessed sleep apnea, n (%)	6 (42.85)
Scoliosis, n (%)	4 (28.57)
Kyphoscoliosis, n (%)	3 (21.42)
Cardiac abnormalities, n (%)	9 (64.28)
Patients with adenoidal hypertrophy, n (%)	9 (81.81)
Adenotonsillectomy, n (%)	8 (66.66)
Tracheostomy, n (%)	0 (0)

The PSG studies revealed the following sleep architecture and respiratory parameters. The mean (range) sleep efficiency was 68.9% (43.6%-93.9%), with a mean (range) sleep latency of 54.76 minutes (0-154.5 minutes) and a mean (range) REM latency of 124.4 minutes (35.5-296.5 minutes). WASO averaged 98.8 minutes (10.6-236.5 minutes), and the mean (range) percentage of REM sleep was 17.4% (8.8%-27.7%). Notably, all patients exhibited a paradoxical breathing pattern, and snoring was detected in 13 patients (92.85%). Respiratory indices from the PSG were as follows: the mean (range) AHI was 14.1 events/hour (0.4-41.9), with a mean (range) REM AHI of 38.2 events/hour (1.8-138.2) and a mean (range) OAHI of 13.48 events/hour (0.35-41.6). The mean (range) central apnea index was 0.35 events/hour (0-1.3), and mixed apnea events were minimal, with a mean (range) of 0.06 (0-0.4). End-tidal CO₂ measurements were obtained in nine patients due to technical issues, yielding a mean (range) CO₂ level of 48.56 mmHg (41-56), with no patients meeting criteria for hypoventilation. Oxygenation data demonstrated an overall mean average oxygen saturation of 96.06% and a mean minimum oxygen saturation of 81.14%. The subjects spent a mean of 4.02% of total sleep time with SpO₂ <90%. Detailed PSG findings are provided in Table 2.

**Table 2:** Polysomnography data (n=14) PSG: polysomnography, n: number of patients, REM: rapid eye movement, WASO: wakefulness after sleep onset, AHI: apnea-hypopnea index, OAHI: obstructive apnea-hypopnea index, CAI: central apnea index, OSA: obstructive sleep apnea, O_2_: oxygen, TST: total sleep time, CO_2_: carbon dioxide, min: minutes

PSG findings	
Sleep efficiency %, mean (range)	68.9 (43.6 – 93.9)
Sleep latency (min), mean (range)	54.76 (0 – 154.5)
REM latency (min), mean (range)	124.4 (35.5 – 296.5)
WASO (min), mean (range)	98.8 (10.6 – 236.5)
REM %, mean (range)	17.4 (8.8 – 27.7)
Snoring, n (%)	13 (92.85)
Paradoxical breathing, n (%)	14 (100)
AHI mean (range)	14.1 (0.4 – 41.9)
REM AHI mean (range)	38.2 (1.8 – 138.2)
CAI mean (range)	0.35 (0 – 1.3)
Mixed apnea mean (range)	0.06 (0 – 0.4)
OAHI mean (range)	13.48 (0.35 – 41.6)
OSA, n (%)	11 (78.57)
Mild OSA, n (%)	3 (27.27)
Moderate OSA, n (%)	3 (27.27)
Severe OSA, n (%)	5 (45.45)
Average O2 saturation mean (range)	96.06 (90 – 99)
Minimal O2 saturation mean (range)	81.14 (31 – 95)
% TST SpO2 < 90% mean (range)	4.02 (0 – 47.1)
Peak CO2 level mean (range)	48.56 (41 – 56)

Three patients didn’t have OSA. Eleven patients were diagnosed with obstructive sleep apnea based on obstructive apnea hypopnea index (OAHI) of ≥ 1.5 events/hour, with prevalence of 78.57%. Severity of OSA was ranging from mild to severe. OSA was mild in three of 11 (27.27%) patients, moderate in three of 11 (27.27%) patients and severe in five of 11 (45.45%) patients.

Regarding therapeutic interventions, adenotonsillectomy was performed in eight of 12 patients for whom data were available (66.66%), while data were missing for two patients. A subset of patients required non-invasive positive pressure ventilation, and none of the patients underwent tracheostomy.

## Discussion

MPS encompasses a range of disorders with seven distinct types [1]. Our study included MPS types I, IV, and VI, and found a high prevalence of OSA at 78.57%. This result is consistent with findings from other studies. For example, Moreira et al. [13] studied the prevalence of OSA in MPS types I, II, and VI at a reference center in Brazil and reported that 68.9% of participants (31/45) had OSA. Similarly, Nashed et al. [14] evaluated MPS patients at a center in Toronto and found a high OSA prevalence of 64% (seven of 11) across types I, II, IV, and VI. Additionally, Leighton et al. [15] studied 26 patients with MPS and found that 88.4% (23/26) had OSA. Although these studies included different MPS types and variable sample sizes, they all demonstrated a high prevalence of OSA in individuals with MPS, which raises the concern about the significance of this complication in those patients.

Consistent with other studies [4,13-16], the majority of our patients had moderate to severe OSA. This not only underscores the high prevalence of OSA but also highlights the severity of the condition. These findings emphasize the importance of performing overnight PSG in all MPS patients promptly, as early detection and intervention are crucial for mitigating associated complications.

The relationship between the type of MPS and the severity of OSA remains unclear. In our cohort, nine patients with MPS type IV were included; of these, seven had OSA, with five exhibiting moderate-to-severe severity, while two had normal sleep study results. In contrast, all patients with MPS type VI demonstrated severe OSA, and patients with MPS type I displayed a variable severity ranging from mild to severe. A systematic review of the literature by Pal et al. [4], which included 294 MPS participants, found that MPS type I had the highest prevalence and severity of OSA. However, the heterogeneity among MPS types may affect the generalizability of these findings.

SDB covers a spectrum ranging from simple snoring to increased airway resistance, progressing to OSA. In our study, we assessed both the clinical history and PSG findings for snoring. Based on clinical history, 35.7% of subjects reported snoring alone, while 42.85% experienced both snoring and witnessed sleep apnea. Interestingly, PSG analysis detected snoring in nearly all patients (92.85%). Moreover, all patients exhibited features of increased upper airway resistance, as evidenced by paradoxical thoracoabdominal movements during PSG. These findings are in agreement with those reported by John et al. [17], who observed snoring in 89.3% and paradoxical breathing in 92.8% of 28 patients with MPS VI, and by Lin et al. [16], who reported 100% snoring prevalence in their cohort of 24 MPS patients. None of our patients had significant central or mixed apnea, and this was comparable to John et al. [17] who found that most of the respiratory events were obstructive.

SDB has significant co-morbidities, if left untreated, it can lead to pulmonary hypertension, arrhythmia, and heart failure. None of our patients had pulmonary hypertension. However, 64.28% (nine of 14) had other cardiac manifestations that might be related to MPS including thickened mitral valve leaflet, mitral valve insufficiency, mitral valve prolapses, thickened aortic valve with stenosis, and mild mitral valve stenosis. One of our patients had atrioventricular block. We suggest that every MPS patient should have a screening ECG and echocardiogram, especially that there is a high prevalence of cardiovascular disease in those patients, and it is considered a significant cause of early mortality [18].

This study aimed to address a prevalent and significant problem in MPS patients. However, there are some limitations: the sample size was small, and not all types of MPS were included, which can be attributed to the rarity of the condition. Additionally, we did not assess the effects of various therapeutic interventions (such as adenotonsillectomy, non-invasive positive pressure ventilation, and enzyme replacement therapy) on SDB. Further research is needed to explore the potential impact of these interventions. Overall, our findings align with other studies that report a high prevalence of SDB among MPS patients.

## Conclusions

Our study demonstrates a high prevalence and significant severity of OSA in our population, consistent with findings from other studies. We recommend that overnight PSG be performed in all patients following diagnosis, as early recognition and timely intervention are crucial to improving quality of life and mitigating the complications associated with this disease. Furthermore, additional studies are needed to examine the potential impact of therapeutic interventions on SDB in this patient population.
